# Extraction Optimization, Purification and Physicochemical Properties of Polysaccharides from *Gynura medica*

**DOI:** 10.3390/molecules21040397

**Published:** 2016-03-23

**Authors:** Fengwei Li, Jian Gao, Feng Xue, Xiaohong Yu, Tao Shao

**Affiliations:** 1 College of Prataculture Science, Nanjing Agricultural University, Nanjing 210095, China; lifengwei1980@126.com; 2 School of Marine and Bioengineering, Yan Cheng Institute of Technology, Yan Cheng 224051, China; gaojian@ycit.cn (J.G.); coldsun_xf@163.com (F.X.); yxh1127@163.com (X.Y.)

**Keywords:** *Gynura medica*, extraction optimization, purification, polysaccharides, activity

## Abstract

Extraction of polysaccharides from *Gynura medica* (GMPs) was optimized by response surface methodology (RSM). A central composition design including three parameters, namely extraction temperature (X_1_), ratio of water to raw material (X_2_) and extraction time (X_3_), was used. The best conditions were extraction temperature of 91.7 °C, extraction time of 4.06 h and ratio of water to raw material of 29.1 mL/g. Under the optimized conditions, the yield of GMPs was 5.56%, which was similar to the predicted polysaccharides yield of 5.66%. A fraction named GMP-1 was obtained after isolation and purification by DEAE-52 and Sephadex G-100 gel chromatography, respectively. GMP-1, with a molecular weight of 401 kDa, mainly consisted of galacturonic acid (GalA), xylose (Xyl), glucose (Glu). Infrared spectroscopy was used to characterize the major functional groups of GMP-1 and the results indicated that it was an acidic polysaccharide. The antioxidant and α-glucosidase inhibitory activities of GMPs and GMP-1 were determined *in vitro*. The results indicated that GMPs and GMP-1 show potential for use in functional foods or medicines.

## 1. Introduction

*Gynura medica* is a recently discovered plant species belonging to the genus Gynura, which is a plant typically cultivated in the southeast region of China. As a natural medicinal plant, *Gynura medica* is often brewed to make a tea to maintain good health, and has the advantage of low toxicity and thus being safe for human consumption [1]. Many studies have shown that alcoholic and aqueous extracts of *Gynura medica* are effective in reducing blood glucose levels, improving oral glucose tolerance and blood serum insulin, that they exhibit good hypoglycemic activity in diabetic animal models [2]. Polysaccharides, which are a family of polymeric carbohydrates that contain 10 or more monosaccharide subunits connected via glycosidic linkages, are the main constituents of *Gynura medica*. It has been reported that polysaccharides extracted from plants have both antioxidant and hypoglycemic biological activity [3,4]. α-Glucosidase inhibitors are a relatively important category of drug used for the treatment of diabetes [5]. As some α-glucosidase inhibitor drugs are known to have toxic side effects, there is growing interest in finding safer α-glucosidase inhibitors from traditional Chinese medicines or natural products from China. Previous research on polysaccharides extracted from *Coriolus versicolor* LH1 mycelia, and *Acacia tortilis* gum exudate which are able to inhibit α-glucosidase have been reported [6,7], but the polysaccharides of *Gynura medica* have not been studied in this respect.

RSM is an effective statistical technique for optimizing complex processes [8]. The RSM technique has advantages over other statistical techniques in that it is less laborious and time-consuming, and produces more precise and statistically significant results for trials using small data sets [9,10]. Many researchers have successfully used this technique to optimize polysaccharide extraction processes [11,12], but to the best of our knowledge, there are no reports examining the extraction of polysaccharides from *Gynura medica*.

In the present study, RSM was used to optimize the extraction of polysaccharides from *Gynura medica* for maximum yield. The extraction parameters were temperature, time and ratio of water to raw material. GMP-1 was then purified and preliminary characterization carried out. The antioxidant and α-glucosidase inhibitory activities of the *Gynura medica* polysaccharides were evaluated too.

## 2. Results and Discussion

### 2.1. Single-Factor Experimental Analysis

**Figure 1:**
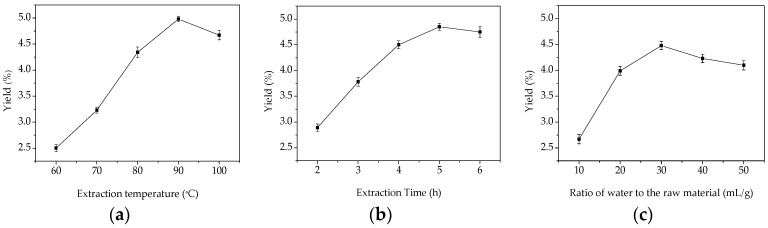
Effects of different extraction parameters on GMPs extraction yield. (**a**) extraction temperature, °C; (**b**) extraction time, h; (**c**) ratio of water to raw material, mL/g.

Extraction temperature is one of the most important variables affecting the extraction yield of GMPs. To study the effect of different temperatures on the yield of GMPs, the extraction process was carried out using different temperatures between 60 and 100 °C. The extraction time was fixed at 3 h, and the ratio of water to the raw material was fixed at 30:1 (mL/g). As shown in Figure 1a, the maximum yield of GMPs was 4.95% when the extraction temperature was 90 °C, and at the highest temperature tested (100 °C) the yield decreased. One explanation for this is that increasing the temperature accelerates solvent diffusion velocity and enhances the solubility of the active ingredients [13,14]. When temperature was 100 °C, however, the extraction yield of GMPs decreased which is probably because the higher temperature destroys the structure of the polysaccharides [15,16]. Considering the extraction yield of GMPs, the results indicate that the high temperature needed to maximize the extract will increase the cost for the extraction process [17], so from and an industrial point of view, temperature ranges from 85 to 95 °C were selected as the optimal conditions in this work.

Extraction time is another factor that influences the extraction efficiency. Extraction time was set between 2 and 6 h, while other extraction parameters were fixed to an extraction temperature 80 °C, and a ratio of water to raw material of 30:1 (mL/g). The effect of extraction time on the yield of GMPs is shown in Figure 1b. When extraction time varied from 2 to 5 h, the extraction efficiency increased and yield reached a maximum of 4.85% at 5 h; however, after this point (5 h), it began to decrease slightly. The lower yield at an extraction time of 6 h might be attributed to the hydrolysis of polysaccharides, which can be hydrolyzed at higher temperatures with long extraction times. Considering the energy savings to be gained and the lower yield with the longest extraction time, the optimal time range for extraction is between 3 and 5 h.

To investigate the effect of the solid material to water volume ratio on the extraction yield of GMPs, the ratio of water to raw material was set at 10:1, 20:1, 30:1, 40:1, and 50:1 mL/g. The other parameters were fixed at an extraction temperature of 80 °C and an extraction time of 3 h. As shown in Figure 1c, the extraction efficiency increased up to a maximum GMPs yield of 4.48% when the ratio was 30:1. The yield decreased when the ratio was increased to 40:1 or 50:1. This is similar to the polysaccharide extraction described in reference [18]. Based on the results, the optimal ratio of water to the raw material is between 20:1 and 40:1.

### 2.2. Optimization of the GMPs Extraction Conditions

#### 2.2.1. Predicted Model and Statistical Analysis

The design matrix and corresponding results of the RSM experiments used to determine the effect of the three variables on the GMPs yield are given in Table 1.

The response and test variables were fitted to Equation (1), and the predicted model was expressed by the quadratic polynomial of Equation (2):

(1) $$Y=A_{0}+\sum_{i=1}^{3}A_{i}X_{i}+\sum_{i=1}^{3}A_{i}{}_{i}X_{i}^{2}+\sum_{i=1}^{2}\sum_{j=i+1}^{3}A_{ij}X_{ij}$$

Y = 5.62 + 0.2X_1_ − 0.053X_2_ + 0.038X_3_ + 0.09X_1_X_2_ − 0.075X_1_X_3_ − 0.087X_2_X_3_ − 0.26X_1_^2^ − 0.16X_2_^2^ − 0.15X_3_^2^ (2)

where in Equation (1) *Y* is the predicted response; *A*_0_ is constant; *A_i_*, *A_ii_*, and *A_ij_* are coefficients; and *X_i_* and *X_j_* are the independent variables X_1_, X_2_, X_3_ are the extraction temperature, ratio of water to the raw material, extraction time, and in Equation (2) Y is the GMPs percentage yield, respectively. Statistical analysis of the model was performed using analysis of variance (ANOVA). Table 2 shows the ANOVA results for the fitted quadratic polynomial model of GMPs extraction. The p values were used as a tool to check the significance of each coefficient, which in turn, may indicate interactions between the variables. The model F value of 25.01 implies the model to be significant (*p* < 0.001). The lack of fit (*p* = 0.222) suggested that it is an adequate model to accurately predict the response variable. The coefficient *R*^2^ = 0.9575 also indicates that the resulting model to be a good fit for GMPs extraction. The significance of each coefficient was determined using F and *p* values. The independent variable X_1_ and three quadratic terms (X_1_, X_2_ and X_3_) significantly affected polysaccharides yield, and the interactions between X_1_ and X_2_, and X_2_ and X_3_ were significant. The empirical model was converted to three-dimensional and contour plots to predict the relationships between the independent variables and the response.

#### 2.2.2. Optimization of Extraction Procedure

**Figure 2:**
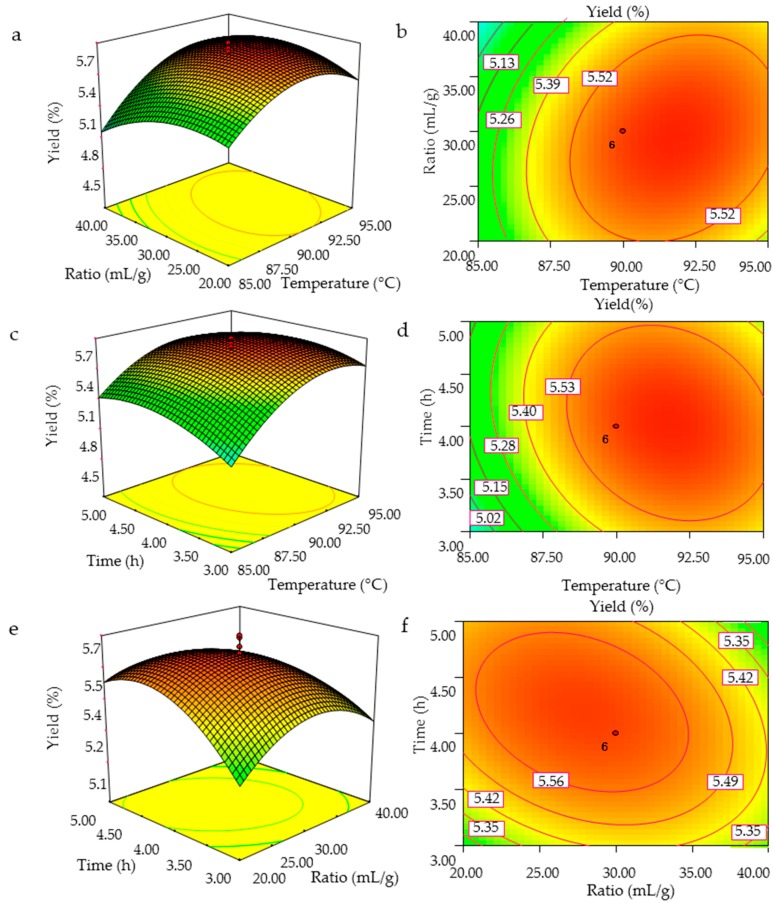
3D response surface plots (**a**,**c**,**e**) and contour plots (**b**,**d**,**f**) showing the interaction effects on the GMPs extraction yield; (**a**,**b**), temperature and ratio of water to the raw material; (**c**,**d**), temperature and extraction time; (**e**,**f**), ratio of water to the raw material and extraction time.

The three-dimensional response surface curves and the contour plots were obtained using the Design-Expert 7.0 software (Stat-Ease Inc., Minneapolis, MN, USA), and the GMPs extraction efficiency as affected by the independent variables (extraction temperature, ratio of water to raw material, and extraction time) and their interactions are shown in Figure 2. The shapes of the contour plots, circular or elliptical, indicate whether the mutual interactions between the variables are significant [19]. The surface plots and contour plots were obtained by varying the values of two test variables with the third variable set at a fixed value. The interactive effects of extraction temperature and ratio of water to the raw material on GMPs yield with the extraction time fixed at 4 h (level 0) are shown in Figure 2a,b. The GMPs yield increased with an increase in extraction temperature and ratio of water to the raw material with optimal values between 85 and 91.01 °C and 20:1 and 28:1 mL/g, respectively, after which the yield decreased. Figure 2b presents a full elliptic contour, which is consistent with the analytical results of regression equation coefficients in Table 2.

Figure 2c,d show the effect of extraction temperature and time on GMPs yield with the ratio of water to raw material fixed to 30:1 mL/g (level 0). The GMPs extraction yield increased as the extraction temperature and time increased, and reached maximum levels at temperatures between 85 and 92 °C and times between 3 and 4.1 h, after which the yield decreased. The singular effect of extraction time on polysaccharides yield, however, was negative in its experimental range, as indicated by the minus sign of the coefficient.

Figure 2e,f show the three dimensional and contour plots of varying the ratio of water to raw material and extraction time on GMPs yield with the extraction temperature fixed at 90 °C. The GMPs yield increased with increasing ratio of water to raw material and increasing extraction time, but the yield then decreased as these two variables were increased further. Figure 2f shows that the mutual interaction between ratio of water to raw material and extraction time was significant.

From the analysis of the interactions between variables, two such interactions (extraction temperature and ratio of water to raw material, ratio of water to raw material and extraction time) among the tested variables are significant (*p* < 0.05). By analyzing the plots, the optimized conditions for the highest GMPs yield were: extraction temperature of 91.7 °C, a ratio of water to raw material of 29.3:1, and an extraction time of 4.06 h. Among the three variables, extraction temperature was the most significant factor affecting the GMPs extraction yield, according to the *p* value, and the gradient of slope in the three-dimensional response surface map. The next most significant factor was the ratio of water to raw material, followed finally by the extraction time (Table 2 and Figure 2).

#### 2.2.3. Verification of the Model

We used selected optimal conditions to test the suitability of the model equation for predicting the optimum response values. Using the optimal conditions for GMPs extraction, our yield was 5.56% (± 0.08%) which is similar to the predicted extraction yield. This indicated that the response model was accurate and adequate for the extraction of GMPs.

### 2.3. Chemical Characterization of GMP-1

**Figure 3:**
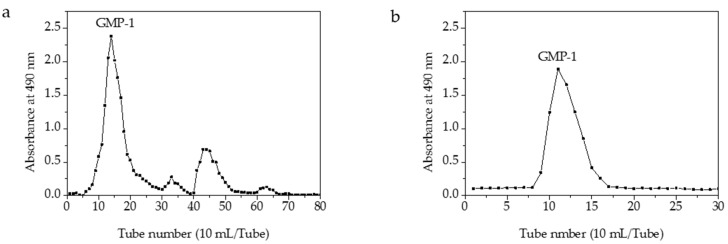
Elution profile of GMPs on a column of DEAE-52 (**a**) and elution profile of GMP-1 on a column of Sephadex G-100 (**b**).

**Figure A1:**
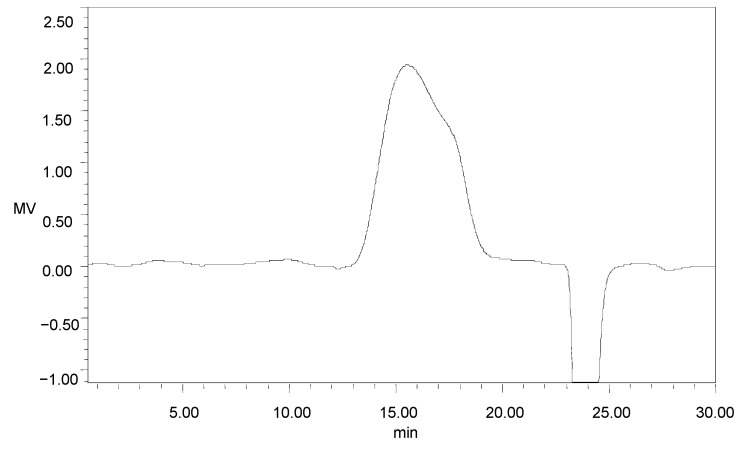
HPGFC profile of GMP-1.

One main elution peak was obtained in the 0.3 M sodium chloride eluent (Figure 3). The HPGFC results indicated the molecular weight of GMP-1was 401 kDa and with purity of 100% (Figure A1).

**Figure 4:**
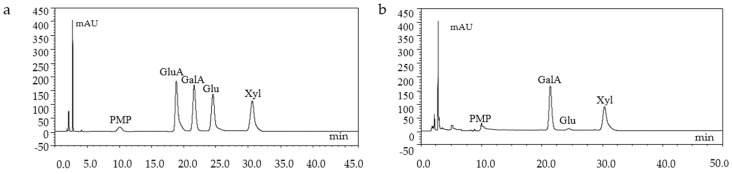
HPLC chromatograms of monosaccharide standards (**a**) and GMP-1(**b**).

The monosachharide composition of GMP-1 was analyzed by HPLC, which indicated GMP-1 was mainly composed of Gal, Xyl and Glu (Figure 4).

**Figure 5:**
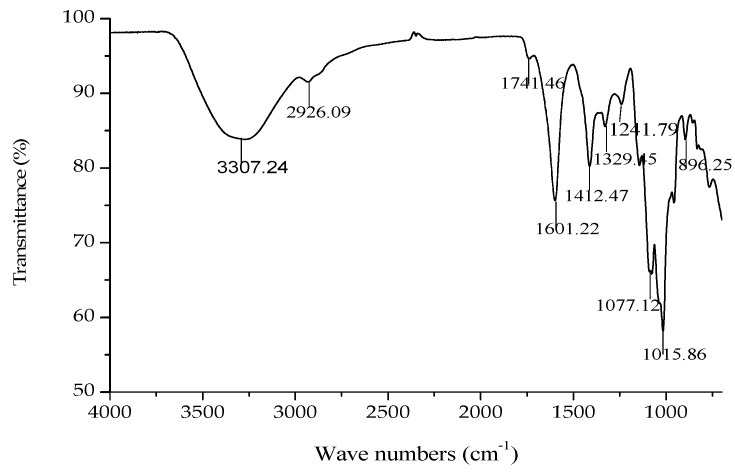
IR spectrum of GMP-1.

The IR spectrum of GMP-1 is shown in Figure 5. A broad stretching peak at 3307.24 cm^−1^ was attributed to the hydroxyl group stretching vibration (–OH) [20]. A weak band at around 2926.09 cm^−1^ was attributed to the C–H stretching vibration. 

Bands at 1741.46 cm^−1^ and 1601.22 cm^−1^ were attributed to the ester carbonyl groups (–C=O) and the carboxylate ion stretching band (COO–) [21]. The absorptions around 1741.46, 1601.22 and 1412.47 cm^−1^ in the IR spectra indicated the presence of uronic acids, which suggested that GMP-1 was a uronic acid-rich polysaccharide. In addition, two stretching peaks at 1077.12 and 1015.86 cm^−1^ indicated overlapped ring vibrations (C–O–C and C–OH) [22]. The peaks at 955.22 and 896.25 cm^−1^ were due to finger print regions for carbohydrates. The band at 896.25 cm^−1^ was the characteristic “anomeric region” absorption of the α-linkage of pyranose and the one at 955.22 cm^−1^ corresponded to the β-linkage of pyranose.

### 2.4. Results of α-Glycosidase Inhibitory Activities

**Figure 6:**
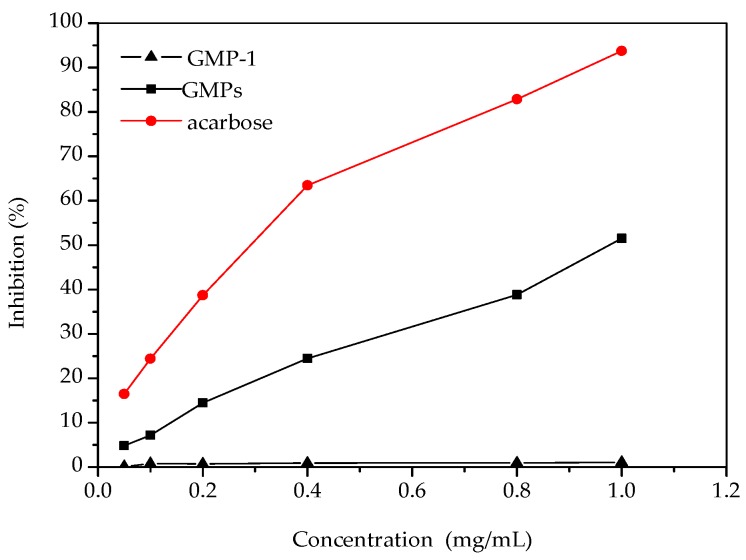
Inhibition effects of samples on α-glucosidase activities.

Figure 6 shows the inhibitory effect of the samples on α-glucosidase activity. The results showed that α-glucosidase inhibition increased with the increase of GMPs concentration. Both GMPs and acarbose showed a dose-dependent inhibitory effect on α-glucosidase, but the potency of the GMPs inhibition was lower than that of acarbose. Previous reports have shown that polysaccharides from a wide range of food or medical plant sources can be effective α-glucosidase inhibitors [23]. Compared with some polysaccharides, GMPs appear to have a higher inhibitory effect on α-glucosidase activity [24], but GMP-1 had no inhibitory effect, even at 1.0 mg/mL. The reason for this may be that the molecular weight of GMP-1 was relatively large compared to that of acarbose, which might affect its interactions with the active sites of α-glucosidase [25]. The results can serve as a guide for new molecular modification of GMP-1.

### 2.5. In Vitro Antioxidant Activity

**Figure 7:**
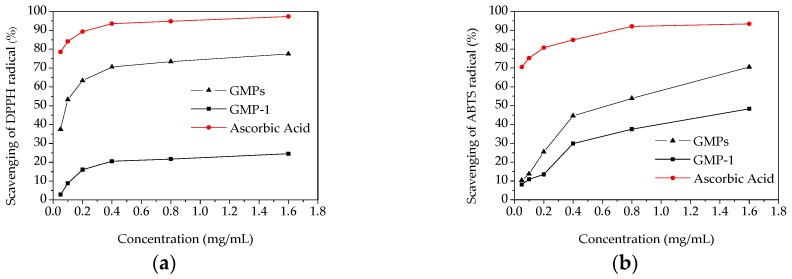
*In vitro* antioxidant activities of GMPs and GPM-1. (**a**) DPPH radical scavenging activity; (**b**) ABTS radical scavenging activity.

The scavenging activity on DPPH radical is a widely used method to evaluate the antioxidant activity of an antioxidant [26]. The scavenging activities of GMPs, GMP-1 and ascorbic acid on DPPH radical were measured, and the results are shown in Figure 7a. A dose-dependent increase in the DPPH radical scavenging capacities was observed. At the concentration of 1.6 mg/mL, the scavenging effects of the GMPs, GMP-1 and ascorbic acid on the DPPH radical were 77.5%, 29.6% and 95.1%, respectively. The scavenging ability of GMPs and GMP-1 were lower than that of ascorbic acid, which is similar to the results of other studies [27]. These results implied that the polysaccharides from *Gynura medica* might be a good resource for obtaining natural antioxidants.

The scavenging effects of samples on ABTS radicals were measured as shown in Figure 7b. Lower antioxidant activities were observed for GMPs and GMP-1 than that found for ascorbic acid. At the highest concentration (1.6 mg/mL), the ABTS radical scavenging activities were 70.46% and 48.34% for GMPs and GMP-1, respectively.

## 3. Materials and Methods

### 3.1. Materials and Reagents

*Gynura medica* stems and leaves were purchased in the Huoshan District, Anhui Province, China. Standard monosaccharides, 1,1-diphenyl-2-picrylhydrazyl (DPPH), 2,2′-azino-bis(3-ethylenebenzothiazoline-6-sulfonic acid) diammonium salt (ABTS), α-glucosidase from baker’s yeast and *p*-nitrophenyl-α-d-glucopyranoside (PNPG) were purchased from Sigma-Aldrich Co. LLC (Shanghai, China). Ethanol, phenol, sulfuric acid and other chemicals and solvents were analytical grade and were purchased from Sinopharm Chemical Reagent Co. (Shanghai, China). DEAE-52 and Sephadex G-100 were purchased from GE Healthcare (Pittsburgh, PA, USA). Acarbose was purchased from Bayer (Leverkusen, Germany).

### 3.2. Extraction of Polysaccharides

The dried *Gynura medica* samples were ground in a lab grinder, passed through a 60 mesh sieve and stored in a sealed polyethylene bag before use. The powdered samples were degreased with petroleum ether (boiling point: 60–90 °C) in a Soxhlet apparatus for 24 h and then extracted with anhydrous alcohol for 24 h to remove some colored materials, lipids, monosaccharides, oligosaccharides and some small molecule materials [28]. The residue was collected by centrifugation, and then dried in an oven at 60 °C to a constant weight. The dried powder (1.00 g) was extracted with varying volumes of water in a flask immersed in a water-bath that was kept at varying temperatures, over varying extraction time periods [29]. After centrifugation, the supernatant was collected and concentrated to 40 mL at 50 °C under reduced pressure [30]. The product was precipitated from the supernatant by addition of 95% alcohol to a final concentration of 80% (*v*/*v*) and then left to stand for 24 h at 4 °C, before the resulting precipitate was collected by centrifugation [31]. After washing three times with anhydrous alcohol, the precipitate was collected and dissolved in distilled water to an appropriate volume. The polysaccharide content was determined by the phenol-sulfuric acid method [32]. Glucose was used as a standard, and the results are expressed as glucose equivalents. The yield (%) of GMPs was calculated as Yield (%) = [the polysaccharides contents of extraction (g)/*Gynura medica* powder weight (g)] × 100%.

### 3.3. Experimental Design

After determining the preliminary range of the extraction variables through single-factor tests, a three-factor-three level central composition design was employed to determine which combination of extraction variables would yield the largest amount of GMPs [33]. Three independent variables—extraction temperature (X_1_), ratio of water to raw material (X_2_) and extraction time (X_3_)—were investigated by using a central composition design (CCD). The ranges of the independent variables and their levels are presented in Table 3.

The response function (Y) was the total extraction yield of GMPs (%). A total of 20 runs were performed, including eight factorial points (coded levels as (+1) and (−1)), and six center points (coded as 0) are shown in Table 1. Statistical analysis was performed with the software package Design-Expert version 7.0. The variables were coded according to Equation (3):

(3) $$Xi=\frac{x_{i}-x_{0}}{\Delta x}i=1,2,3$$

where X*i* is the coded value of independent variable, *x_i_* is the actual value of the independent variable, *x*_0_ is the actual value of independent variable at the center point, and Δ*x* is the step change of the variable. 

### 3.4. Purification of Crude Polysaccharides

The crude GMPs were obtained under optimal conditions and dissolved in distilled water. Pigments were removed by D101 macroporous resin according to a previous method [34]. Proteins were removed by using the Sevage and enzymatic methods. The GMPs were then redissolved in distilled water and further separated by DEAE-52 and Sephadex G-100 column chromatography. The DEAE-52 column was eluted in a stepwise manner with deionized water, 0.1, 0.2, 0.3 and 0.5 M sodium chloride. Fractions of 10 mL each were collected using an automatic fraction collector. The carbohydrates were determined by the phenol-sulfuric acid method using glucose as the standard, and the concentration of the main peak was convergent [35]. The collected polysaccharides were loaded onto a Sephadex G-100 column, which was eluted with deionized water. The main fractions were assayed by the phenol-sulfuric acid method. After dialysis and lyophilization, the collected fraction was named GMP-1.

### 3.5. Chemical Characterization of GMP-1

The molecular mass of GMP-1 was determined by the high performance gel-filtration chromatography (HPGFC) [36]. The analysis was performed on a Waters HPLC apparatus (Waters Co., Milford, MA, USA) equipped with an ultra-hydrogel linear column (300 mm × 7.8 mm) and a refractive index detector (RID). The column was eluted with 0.1 M NaNO_3_ with a flow rate of 0.9 mL/min.

The monosaccharide composition was performed using a HPLC system (UltiMate 3000, Thermo Fisher Scientific Inc., Sunnyvale, CA, USA) after pre-column derivatization according to a previously method with some modifications [37]. The sample (5 mg) was hydrolyzed with 2 M trifluoroacetic acid (TFA, 2.5 mL) at 110 °C for 6 h. Then, the excess TFA was removed by evaporation with methanol. Samples were derivatized with 0.3 M NaOH (0.5 mL) and 0.5 M 1-phenyl-3-methyl-5-pyrazolone (PMP, 0.2 mL) at 70 °C for 30 min. After cooled, samples were neutralized with HCl and excess PMP was extracted three times with isoamyl acetate (1 mL). The aqueous layer was filtered through a 0.22 µm membrane for HPLC analysis. The analytical column was ODS-C_18_ (150 mm × 4.6 mm, 5 µm), monitored at 250 nm. The mobile phase was a mixture of acetate buffer (0.1 M) and acetonitrile in the ratio of 81:19 (*v*/*v*) at a flow rate of 0.8 mL/min. GMP-1 was ground with KBr powder and then pressed into pellets for IR determination between wave number range of 4000 and 500 cm^−1^. The functional groups were recorded on a NEXUS 670 IR spectrophotometer (Thermo-Nicolet, Waltham, MA, USA).

### 3.6. α-Glucosidase Inhibitory Activity

The α-glucosidase inhibitory activity of the GMPs and GMP-1 were determined according to the method of Ren with minor modifications [38]. Acarbose was used as a positive control. The initial concentration of the enzyme solution was 0.04 U/mL in a phosphate buffer pH 6.8. The initial concentration of the PNPG was 0.5 mM in the same buffer. A 40 μL solution of α-glucosidase was preincubated in 96 well plates for 5 min at 37 °C with 40 μL of the respective test solution (acarbose and samples at various concentrations). The blank solution had phosphate buffer in place of the sample solution. The enzymatic reaction was initiated by adding 20 μL of PNPG, and the mixture was incubated for another 30 min at 37 °C. The reaction was terminated by adding 100 μL of Na_2_CO_3_ (0.1 M, pH 9.8). After incubation, the UV absorbance was recorded at 405 nm using a microplate reader. The α-glucosidase inhibitory activity was calculated using Equation (4):

Inhibition ratio (%) = 100% × [OD_blank_ − (OD_sample_ − OD_background_)]/OD_blank_ (4)

### 3.7. In Vitro Antioxidant Activity

#### 3.7.1. DPPH Radical Scavenging Assay

The DPPH radical scavenging capacity was assessed according to the method described by Ganesh *et al.* [39] with some modifications. Three mL solution samples were taken at different concentrations. Then, 3.0 mL 0.1 mM DPPH solution was added, and after homogenisation, the fluid was kept in the dark for 30 min. The mixture solution absorbance was tested at a wavelength of 517 nm. Ascorbic acid was used as a control. The DPPH radical scavenging rate was calculated according to the following Equation (5):

Scavenging rate (%)= [1 − (A_sample_ − A_blank_)/A _blank_] × 100% (5)

where A_blank_ was the absorbance of the system containing 60% ethanol instead of sample.

#### 3.7.2. ABTS Radical Scavenging Assay

Samples were measured according to the method adapted from Smith with some modifications [40]. Briefly, 7 mM ABTS solution in ethanol was added to an equal volume of 2.45 mM potassium persulfate. The mixture was kept in the dark at room temperature for 16 h and then diluted with ethanol to an absorbance of 0.7 at 734 nm before use. Next, 0.5 mL samples at various concentrations were mixed with 4.5 mL of ABTS solution, and the mixture was kept in the dark at room temperature for 30 min. The absorbance was measured at 734 nm against a blank. Ascorbic acid was used as a reference. The ABTS radical scavenging activity was calculated by the following Equation (6):

Scavenging rate (%) = (A_blank_ − A_sample_)/A_blank_ × 100% (6)

where A_blank_ is the absorbance of distilled water mixed with ABTS.

## 4. Conclusions

In the present work, the extraction conditions of polysaccharides from *Gynura medica* were optimized by RSM. The results showed that the independent variable extraction temperature, quadratic terms and interaction effects significantly affected the GMPs yield. The best conditions were: extraction temperature 91.7 °C, extraction time 4.06 h and ratio of water to raw material 29.1 mL/g. Under the optimal conditions, the GMPs were obtained in 5.56% yield. An acid-rich polysaccharide GMP-1 with molecular weight of 401 kDa was obtained after isolation and purification by DEAE-52 and Sephadex G-100 gel chromatography. Preliminary *in vitro* tests indicated that the GMPs were able to inhibit the activity of α-glucosidase. Both GMPs and GMP-1 showed strong antioxidant activities on DPPH and ABTS radicals. Further work on purification and structure of GMPs is in progress.

## Figures and Tables

**Table 1:** Central composition design matrix and the response values for GMPs yield.

Run	X_1_	X_2_	X_3_	Yield (%)
1	90.0	30.0	4.0	5.62
2	95.0	40.0	3.0	5.33
3	81.59	30.0	4.0	4.52
4	90.0	30.0	4.0	5.65
5	90.0	30.0	4.0	5.49
6	85.0	20.0	5.0	5.30
7	95.0	20.0	5.0	5.29
8	95.0	20.0	3.0	5.25
9	85.0	20.0	3.0	4.83
10	85.0	40.0	5.0	4.67
11	90.0	30.0	4.0	5.70
12	90.0	30.0	2.32	5.11
13	90.0	13.18	4.0	5.12
14	90.0	30.0	5.68	5.23
15	85.0	40.0	3.0	4.68
16	98.41	30.0	4.0	5.19
17	90.0	46.82	4.0	5.48
18	90.0	30.0	4.0	5.57
19	95.0	40.0	5.0	5.15
20	90.0	30.0	4.0	5.69

**Table 2:** Analysis of variance for the response surface quadratic model.

Source	Sum of Squares	Degrees of Freedom	Mean Square	*f* Value	*p* Value (Prob. > F)
Model	2.18	9	0.24	25.01	<0.0001 ^a^
X_1_	0.52	1	0.52	53.01	<0.0001 ^a^
X_2_	0.038	1	0.038	3.94	0.0753
X_3_	0.020	1	0.020	2.06	0.1821
X_1_X_2_	0.065	1	0.065	6.68	0.0272 ^b^
X_1_X_3_	0.045	1	0.045	4.64	0.0566
X_2_X_3_	0.061	1	0.061	6.32	0.0307 ^b^
X_1_^2^	1.00	1	1.00	102.63	<0.0001 ^a^
X_2_^2^	0.35	1	0.35	36.50	0.0001 ^a^
X_3_^2^	0.33	1	0.33	34.07	0.0002 ^a^
Residual	0.097	10	9.696 × 10^−3^		
Lack of Fit	0.065	5	0.013	2.07	0.2220 ^ns^
Pure Error	0.032	5	6.320 × 10^−3^		
Cor. Total	2.28	19			
*R*^2^	0.9575				
Adj. *R*^2^	0.9192				
Pred. *R*^2^	0.7565				
Adeq. precision	15.391				
C.V.%	1.88				

^ns^ not significant; ^a^. Significant at *p* < 0.001; ^b^. Significant at *p* < 0.05.

**Table 3:** Independent variables and their levels used in the response surface design.

Independent Variables	Level				
	−1.682	−1	0	1	1.682
Extraction temperature (X_1_, °C)	81.59	85	90	95	98.41
Ratio of water to the raw material (X_2_, mL/g)	13.18	20	30	40	46.82
Extraction time (X_3_, h)	2.318	3	4	5	5.682
